# ZBP1 phosphorylation at serine 181 regulates its dendritic transport and the development of dendritic trees of hippocampal neurons

**DOI:** 10.1038/s41598-017-01963-2

**Published:** 2017-05-12

**Authors:** Anna S. Urbanska, Aleksandra Janusz-Kaminska, Katarzyna Switon, Alicia L. Hawthorne, Malgorzata Perycz, Malgorzata Urbanska, Gary J. Bassell, Jacek Jaworski

**Affiliations:** 1 International Institute of Molecular and Cell Biology, Warsaw, Poland; 2 Department of Cell Biology, Emory University School of Medicine, Atlanta, GA USA

## Abstract

Local protein synthesis occurs in axons and dendrites of neurons, enabling fast and spatially restricted responses to a dynamically changing extracellular environment. Prior to local translation, mRNA that is to be translated is packed into ribonucleoprotein particles (RNPs) where RNA binding proteins ensure mRNA silencing and provide a link to molecular motors. ZBP1 is a component of RNP transport particles and is known for its role in the local translation of β-actin mRNA. Its binding to mRNA is regulated by tyrosine 396 phosphorylation, and this particular modification was shown to be vital for axonal growth and dendritic branching. Recently, additional phosphorylation of ZBP1 at serine 181 (Ser181) was described in non-neuronal cells. In the present study, we found that ZBP1 is also phosphorylated at Ser181 in neurons in a mammalian/mechanistic target of rapamycin complex 2-, Src kinase-, and mRNA binding-dependent manner. Furthermore, Ser181 ZBP1 phosphorylation was essential for the proper dendritic branching of hippocampal neurons that were cultured *in vitro* and for the proper ZBP1 dendritic distribution and motility.

## Introduction

Local protein synthesis is a phenomenon of regulated translation that occurs in subdomains of a cell, often distant from the major sites of protein production (e.g., leading edges of fibroblasts and neuronal axonal growth cones and dendrites). It provides a means of local and transient cell polarization and enables fast responses to local stimuli. Neurons have been the most intensely studied because of their importance for local protein synthesis. In these cells, local protein synthesis regulates axonal growth cone navigation, dendritic arbor and spine formation, and synaptic plasticity^1–3^. Local translation requires the transportation of translationally dormant mRNA to the destination point, often over relatively long distances, where mRNA becomes derepressed and translationally competent. mRNAs that are to be locally synthesized contain tagging sequences that are recognized and bound by protein complexes that provide both translational silencing and attachment to molecular motors (i.e., kinesins, dyneins, and myosins). These so-called ribonucleoprotein particles (RNPs) can be transported along microtubules and microfilaments, or they may stall at destination points while awaiting a signal to release bound mRNA for translation^4^. In neurons, changes in RNP dynamics and the translational unsilencing of mRNA can occur very rapidly, often in response to extracellular stimuli (e.g., trophic factors, calcium influx, or neurotransmitters) and are controlled by the phosphorylation of RNP proteins^5–7^.

Zipcode binding protein 1 (ZBP1; also known as IMP1 or IGF2BP1) is an mRNA binding protein that is found in RNPs. ZBP1 was described as β-actin mRNA-binding protein, which ensures its proper cellular distribution and translational silencing. In neurons, the local synthesis of β-actin, controlled by ZBP1 and its orthologs (e.g., IMP1 and Vg1 RBP), contributes to axonal growth cone navigation, proper dendritic arborization, and dendritic spine formation^8–12^. The ability of ZBP1 to silence β-actin mRNA is dynamically regulated by phosphorylation. In response to trophic factors, ZBP1 is phosphorylated at tyrosine 396 (Tyr396) by Src and releases β-actin mRNA for translation^5, 6, 13^. In fact, proper axonal growth cone navigation and dendritic branching both require the ability of ZBP1 to be phosphorylated at Tyr396^5, 6, 9^. Recently, additional phosphorylation sites of ZBP1 and its orthologs were described. For example, in non-neuronal cells, serine 181 (Ser181) of IMP1 is cotranslationally phosphorylated by mammalian/mechanistic target of rapamycin complex 2 (mTORC2) and protected by RNA binding. In mouse embryonic fibroblasts and RD rhabdomyosarcoma cells, Ser181 ZBP1 phosphorylation is needed for proper splicing and the cap-independent translation of insulin growth factor 2 (IGF2) mRNA. However, to date, the presence of Ser181 ZBP1 phosphorylation and its regulation in neurons and significance for neuronal development have not been tested.

The present study showed that ZBP1 is stably phosphorylated at Ser181 in neurons. This phosphorylation was mTORC2- and mRNA binding-dependent. Moreover, we found less ZBP1 with phosphorylated Ser181 in cells with increased Src activity. Furthermore, rescue experiments with ZBP1 short-hairpin RNA (shRNA) and a ZBP1 mutant that mimicked non-phosphorylated ZBP1 (ZBP1_S181A_) provided evidence that Ser181 ZBP1 phosphorylation was critical for the proper dendritic branching of hippocampal neurons that were cultured *in vitro*. Finally, we found that Ser181 phosphorylation is needed for proper ZBP1 mobility and dendritic distribution. Taken together these findings uncover the role of Ser181 phosphorylation in ZBP1 trafficking and dendritic development.

## Results

### ZBP1 is phosphorylated at Ser181 in neurons in an mTORC2-dependent manner

**Figure 1:**
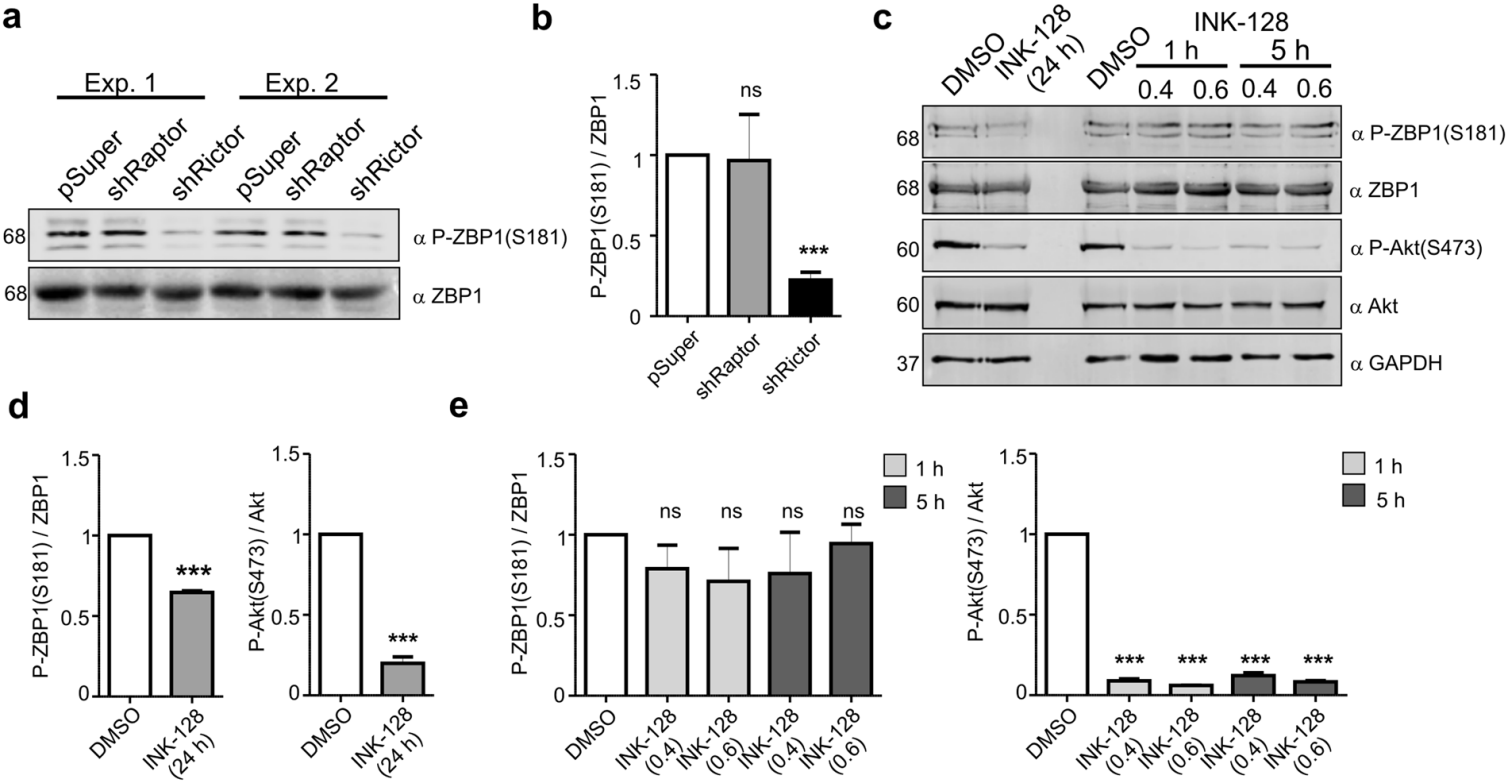
ZBP1 is phosphorylated at Ser181 in neurons in an mTORC2-dependent manner. (**a**) Western blot analysis of levels of ZBP1 phosphorylated at Ser181 (P-ZBP1[S181]) and total ZBP1 in protein lysates obtained from DIV8 neurons nucleofected on DIV0 with empty pSuper (control vector) or pSuper that encoded shRNA against Raptor or Rictor. Cropped blots are presented (for full length blots see Supplementary Information). (**b**) Results of quantitative Western blot analysis. The data are presented as a ratio of the signal intensity of P-ZBP1(S181) to the signal intensity of ZBP1 ± SEM. ***p < 0.001, *ns* - non significant (One sample *t*-test). Number of independent experiments (*N* = 4). (**c**) Western blot analysis of levels of P-ZBP1(S181), total ZBP1, P-Akt(S473) and total Akt in protein lysates obtained from DIV10 neurons treated for 1, 5 or 24 h with vehicle (DMSO) or INK128 at indicated concentrations. GAPDH is shown as an additional loading control. Cropped blots are presented (for full length blots see Supplementary Information). (**d**) Results of quantitative Western blot analysis. The data are presented as a ratio of the signal intensity of P-ZBP1(S181) to ZBP1 or P-Akt to Akt ± SEM. ***p < 0.001, *ns* - non significant (One sample *t*-test). Number of independent experiments (*N* = 3).

ZBP1 is phosphorylated at Ser181 in non-neuronal cells^14^, but this phosphorylation was not previously investigated in neurons. To verify the existence of such phosphorylation in neurons, we used a custom-made antibody that was raised against Ser181-phosphorylated rat ZBP1. This antibody detected, in an alkaline phosphatase-dependent manner, green fluorescent protein (GFP)-tagged wildtype rat ZBP1 (~96 kDa) but not ZBP1 with Ser181 mutated to alanine (S181A) that was overexpressed in Neuro2a cells (Supplementary Fig. S1). Both proteins were recognized by an antibody that detects ZBP1 regardless of its phosphorylation status (Supplementary Fig. S1). The new phospho-specific antibody detected an endogenous protein of a predicted size (~68 kD) in lysates of developing rat neurons that were cultured *in vitro* and lysates of hippocampi and cortices of embryonic day 17 (E17) wildtype mice (Fig. 1a, Supplementary Fig. S1). No such band was detected in protein lysates that were obtained from hippocampi and cortices of E17 *IMP1* ^−/−^ mice^15^ (Supplementary Fig. S1), further supporting the specificity of the tested antibody.

Next, we tested whether mTORC2 contributes to ZBP1 phosphorylation in neurons similarly to the way it does in non-neuronal cells^14^. Freshly isolated rat embryonic cortical neurons were nucleofected with pSuper (control plasmid) or pSuper that encoded previously validated shRNA against Raptor or Rictor (i.e., components of mTORC1 and mTORC2, respectively^16, 17^; Supplementary Fig. S2). The analysis of lysates from 8 days *in vitro* (DIV8) electroporated neurons indeed revealed a decrease in P-ZBP1(S181)/ZBP1 ratio in cells with Rictor knockdown compared with Raptor knockdown and control cells (Fig. 1a,b). To further confirm the role of mTORC2 in controlling Ser181 ZBP1 phosphorylation in neurons and gain further insight into the dynamics of this process, we treated DIV10 neurons with Ink128, an ATP-competitive inhibitor of mTOR, for 1, 5 or 24 h. mTOR inhibition for 24 h was sufficient to decrease Ser181 ZBP1 phosphorylation (Fig. 1c,d). In contrast, shorter treatments resulted in no difference between treated and untreated cells, although both inhibitors led to the loss of Ser473 phosphorylation of Akt, a prototypical mTORC2 substrate (Fig. 1c,e). These results show that mTORC2 contributes to Ser181 ZBP1 phosphorylation, which is rather stable under basal culture conditions.

### Ser181 phosphorylation is regulated in response to Src-dependent ZBP1 modification

**Figure 2:**
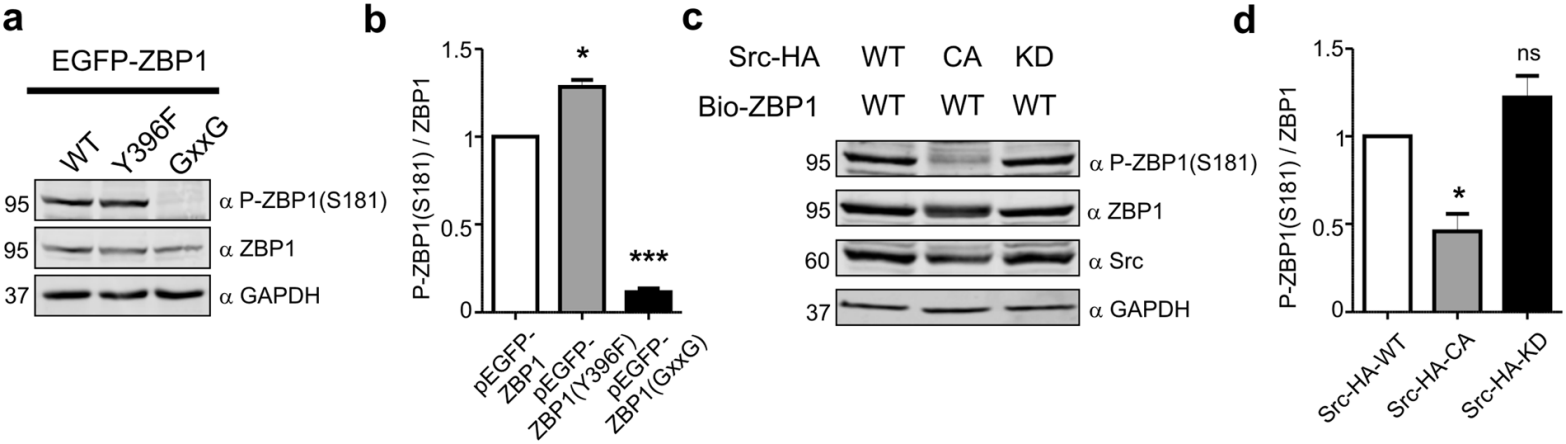
Serine 181 phosphorylation is dynamically regulated by Src activity. (**a)** Western blot analysis of levels of ZBP1 phosphorylated at Ser181 (P-ZBP1[S181]) and total ZBP1 in protein lysates obtained from Neuro2a cells transfected with pEGFP-ZBP1, pEGFP-ZBP1_Y396F_, and pEGFP-ZBP1_GxxG_. GAPDH is shown as an additional loading control. Cropped blots are presented (for full length blots see Supplementary Information). (**b**) Results of quantitative Western blot analysis. The data are presented as a ratio of the signal intensity of P-ZBP1(S181) to the signal intensity of ZBP1 ± SEM. *p < 0.05, ***p < 0.001 (One sample *t*-test). Number of independent experiments (*N* = 3). (**c**) Western blot analysis of levels of ZBP1 phosphorylated at Ser181 (P-ZBP1[S181]), total ZBP1, and Src in protein lysates obtained from Neuro2a cells transfected with pBIO-ZBP1 and plasmids that encoded Src_WT_, Src_CA_, or Src_DN._ GAPDH is shown as an additional loading control. Cropped blots are presented (for full length blots see Supplementary Information). (**d**) Results of quantitative Western blot analysis. The data are presented as a ratio of the signal intensity of P-ZBP1(S181) to the signal intensity of ZBP1 ± SEM. *p < 0.05, *ns* - non significant (One sample *t*-test). Number of independent experiments (*N* = 4).

Next, we tested under what additional conditions the phosphorylation of ZBP1 at Ser181 can change in neural cells. In non-neuronal cells, Ser181 is dephosphorylated upon the release of bound mRNA^14^. Consistent with this finding, a ZBP1 mutant with decreased ability to bind mRNA (ZBP1_GXXG_) was poorly phosphorylated at Ser181 when it was overexpressed in Neuro2a cells compared with wildtype ZBP1 (Fig. 2a,b). In neurons, the release of β-actin mRNA from ZBP1 RNPs is dynamically regulated by the Src-driven phosphorylation of Tyr396^5^. Thus, the ZBP1 mutant, which cannot be phosphorylated by Src (ZBP1_Y396F_), should less efficiently release bound mRNA and be more phosphorylated at Ser181. Indeed, as opposed to ZBP1_GXXG_, ZBP1_Y396F_ was slightly, although significantly, more phosphorylated at Ser181 compared with wildtype protein when it was overexpressed in Neuro2a cells (Fig. 2a,b). The phosphorylation of ZBP1 at Ser181 significantly decreased when ZBP1 was cotransfected to Neuro2a with constitutively active Src kinase but not its kinase-dead variant (Fig. 2c,d). Tyr396 ZBP1 phosphorylation was absent in cells that were transfected with Src^KD^, whereas it was present in cells that overexpressed Src^CA^ (Supplementary Fig. S3). Notably, wildtype and phospho-mutants (S181A, S181E [phospho-mimicking mutant]) of ZBP1 were phosphorylated at Tyr396 by Src to the same extent (Supplementary Fig. S3). This suggests that ZBP1 Src-dependent phosphorylation and subsequent mRNA release may regulate Ser181 phosphorylation. On the other hand, Ser181 phosphorylation does not prevent Tyr396 ZBP1 phosphorylation by Src. Altogether, our observations suggest that Ser181 phosphorylation in neurons is relatively stable but can change in response to Src-dependent ZBP1 phosphorylation.

### ZBP1 Ser181 phosphorylation is critical for the proper dendritic arborization of hippocampal neurons

**Figure 3:**
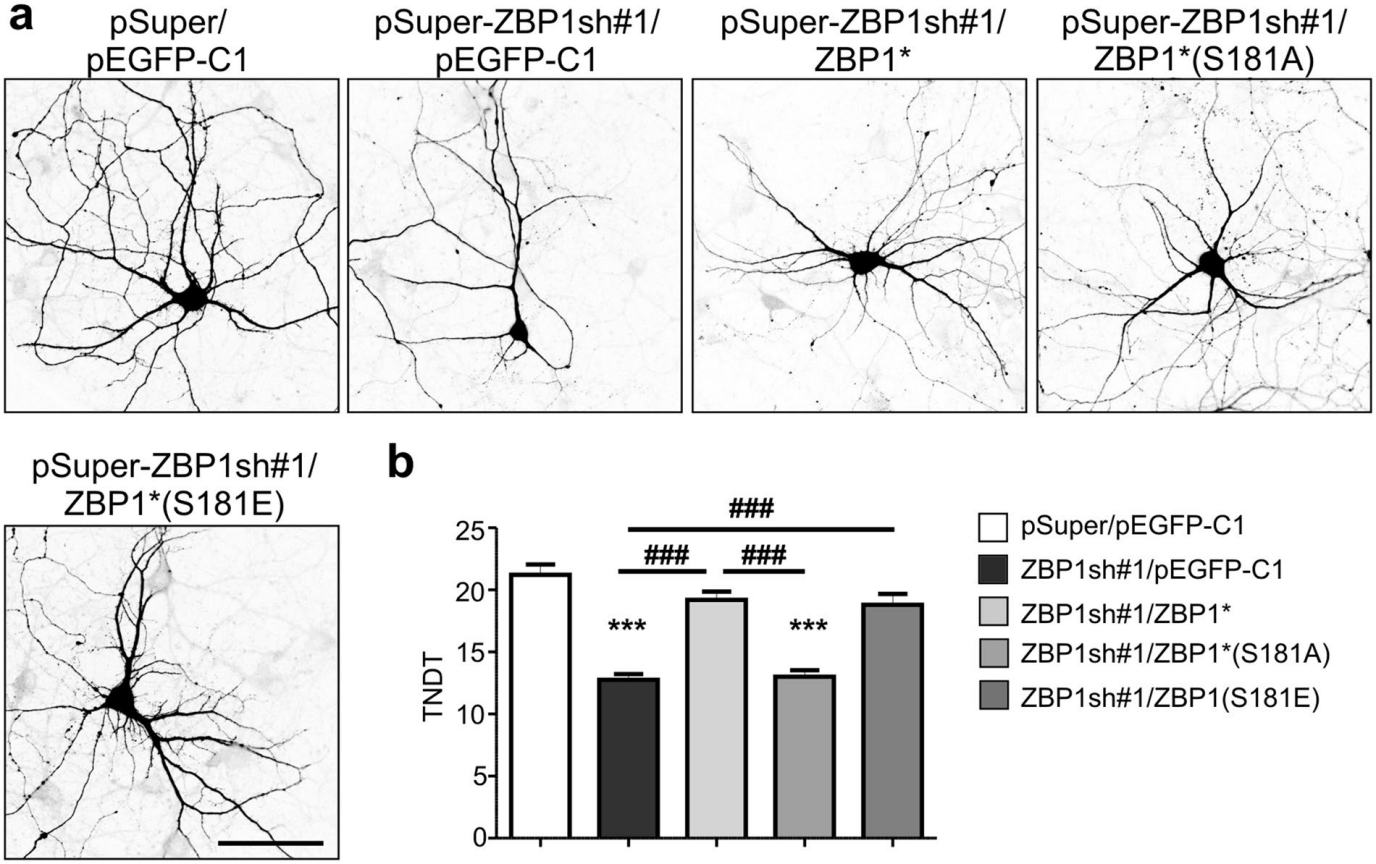
ZBP1 phosphorylation at Ser181 is needed for the proper dendritic arborization of hippocampal neurons. (**a**) Representative confocal images of cultured hippocampal neurons transfected on DIV8 for 2 days as indicated. Neuron morphology was visualized by co-transfection with mRFP. Scale bar = 50 µm. (**b**) Total number of dendritic tips (TNDT) of neurons transfected as in (**a**). The data are expressed as mean ± SEM. ***p < 0.001, compared with pSuper/pEGFP-C1-transfected cells; ^###^ *p* < 0.001, compared as indicated (Kruskal-Wallis test followed by Dunn’s *post hoc* test). Cell images were obtained from two independent culture batches. Number of cells per variant: pSuper/pEGFP-C1 (*n* = 35), ZBP1sh#1/pEGFP-C1 (*n* = 36), ZBP1sh#1/pEGFP-ZBP1* (*n* = 35), ZBP1sh#1/pEGFP-ZBP1*_S181A_ (*n* = 55), and ZBP1sh#1/pEGFP-ZBP1*_S181E_ (*n* = 47).

Next, we investigated whether Ser181 phosphorylation is meaningful for one of the neuronal functions of ZBP1. We previously found that ZBP1 was critical for the proper pattern of dendritic branching of hippocampal neurons that were cultured *in vitro* ^9^. Thus, we examined the contribution of Ser181 ZBP1 phosphorylation to the proper development of dendritic arbors. First, we confirmed in Neuro2a cells that S181 mutations either to A or E do not affect ZBP1 stability (Supplementary Fig. S4). Next, we used the same approach as previously described^9^, in which we assayed ZBP1_S181A_ and ZBP1_S181E_ mutants with regard to their ability to rescue dendritic arbor simplification that was induced by ZBP1 knockdown. DIV7 hippocampal neurons were transfected with a plasmid that encoded validated ZBP1 shRNA (pSuper-ZBP1sh) and a plasmid that encoded an RNA interference-resistant wildtype, S181A or S181E EGFP-ZBP1 mRNA (EGFP-ZBP1*, EGFP-ZBP1*_S181A_, and EGFP-ZBP1*_S181E_). As an additional control, we used cells that were transfected with pSuper-ZBP1sh and EGFP. After 3 days, the total number of dendritic tips (TNDT) was analyzed. As previously reported, an shRNA against ZBP1 resulted in a significant decrease in the TNDT, and co-transfection of EGFP-ZBP1* fully rescued this defect (Fig. 3). However, when EGFP-ZBP1*_S181A_ was transfected instead, we did not observe TNDT rescue. The cotransfection of EGFP-ZBP1*_S181E_ fully rescued the knockdown phenotype but did not cause any further increase in the TNDT. This observation is consistent with a previous suggestion that the majority of cellular ZBP1 is phosphorylated at Ser181^14^. However, when Y396F mutation was introduced to ZBP1_S181E_, the double mutant lost the ability to rescue phenotypic effects of ZBP1 knockdown, similar to ZBP1Y396F (Supplementary Fig. S5). Thus, we concluded that the phosphorylation of ZBP1 at Ser181 is involved in the control of proper neuronal dendritic morphology but its role might be secondary to phosphorylation of tyrosine 396 of ZBP1.

### Ser181 phosphorylation is required for the proper dendritic distribution of ZBP1

**Figure 4:**
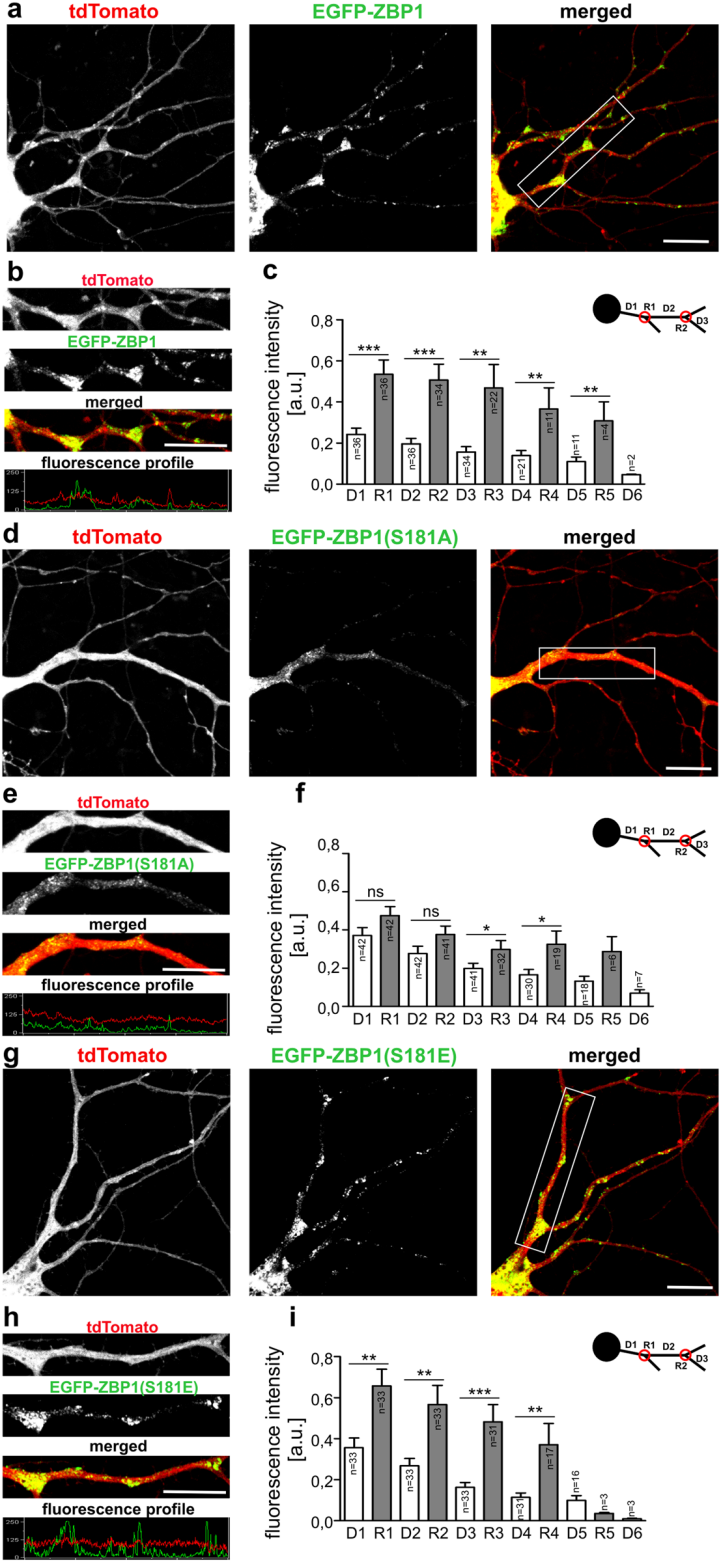
ZBP1 phosphorylation at Ser181 is needed for its proper dendritic distribution. Representative confocal images of hippocampal neurons transfected on DIV7 with pEGFP-ZBP1-P2A-tdTomato (**a**), pEGFP-ZBP1_S181A_-P2A-tdTomato (**d**), or pEGFP-ZBP1_S181E_-P2A-tdTomato (**g**). Expression proceeded for 30 h. (**b**,**e**,**h)** Representative micrographs of single dendrites of transfected cells with corresponding profiles of fluorescence. (**c**,**f**,**i**) Normalized mean intensities of EGFP-ZBP1 fluorescence ± SEM measured at dendritic branching points (R) and interbranching dendritic segments (D). The data were obtained from three independent neuronal cultures. The data are expressed as mean ± SEM. ***p < 0.001, **p < 0.01, *p < 0.05, ns - non-significant. Student’s *t*-test. Number of cells per variant: pEGFP-ZBP1-P2A-tdTomato (*n* = 36), pEGFP-ZBP1(S181A)-P2A-tdTomato (*n* = 42), pEGFP-ZBP1(S181E)-P2A-tdTomato (*n* = 33). Number of analyzed branches for each order is provided in each graph.

In our previous work, we found that ZBP1 is differentially distributed in dendrites of neurons that are actively growing their dendritic arbors compared with mature dendrites. This suggested that proper dendritogenesis requires accurate ZBP1 distribution, namely the concentration of ZBP1 at dendrite branching points, which may correspond to hot spot of local synthesis of β-actin and other proteins needed for dendritic growth and branching. Accordingly, we tested whether Ser181 ZBP1 phosphorylation contributes to the spatial dendritic distribution of this protein. For more accurate quantitative analysis, we designed plasmids that simultaneously expressed EGFP-ZBP1 (or S181A and S181E mutants) and fluorescent protein - tdTomato (Supplementary Fig. S6) to correct for effects of unequal transfection of individual neurons. Next, the spatial distribution of EGFP-ZBP1 and its mutants in dendrites of DIV10 neurons was quantitatively analyzed. Wildtype ZBP1 concentrated at dendritic branching points as described previously^9^ (Fig. 4a). ZBP1_S181E_ displayed a very similar distribution (Fig. 4c). In contrast, ZBP1_S181A_ was almost equally distributed between branching points and interbranching dendritic segments (Fig. 4b). Comparisons of the normalized EGFP fluorescence ratio in a dendritic D2 segment to the following R2 branching point (Fig. 4) of ZBP1- and ZBP1_S181A_-expressing neurons revealed a statistically significant difference (EGFP-ZBP1 [*n* = 34]: 0.53 ± 0.05; EGFP-ZBP1_S181A_ [*n* = 32]: 0.88 ± 0.09; *p* = 0.0014, Mann-Whitney test), confirming that ZBP1_S181A_ was more equally distributed and not enriched at dendritic branch points.

Changed distribution of ZBP1_S181A_ theoretically could be a result of its inability to bind mRNA as it was shown for ZBP1_GXXG_ mutant. Thus, we tested the ability of ZBP1_S181A_ to bind β-actin mRNA, a canonical target of ZBP1. ZBP1_S181A_ transfected to cultured *in vitro* hippocampal neurons of *IMP1* ^−/−^ mice still formed granules, some of which colocalized with β-actin mRNA (Supplementary Fig. S7), suggesting that the capability of ZBP1_S181A_ to bind mRNA could not be completely lost. However, in RNA-IP performed from Neuro2a cells transfected with ZBP1 or ZBP1_S181A_, we observed that S181A substitution resulted in a 50% decrease in the quantity of β-actin mRNA in the ZBP1-bound mRNA pool when compared to wildtype ZBP1 (Supplementary Fig. S7). Altogether, we concluded that while there was less β-actin mRNA bound to ZBP1_S181A_ its RNA binding capacity was not the primary reason of the altered ZBP1_S181A_ distribution in the dendrites.

### Lack of Ser181 phosphorylation affects ZBP1 mobility and KIF5A binding

**Figure 5:**
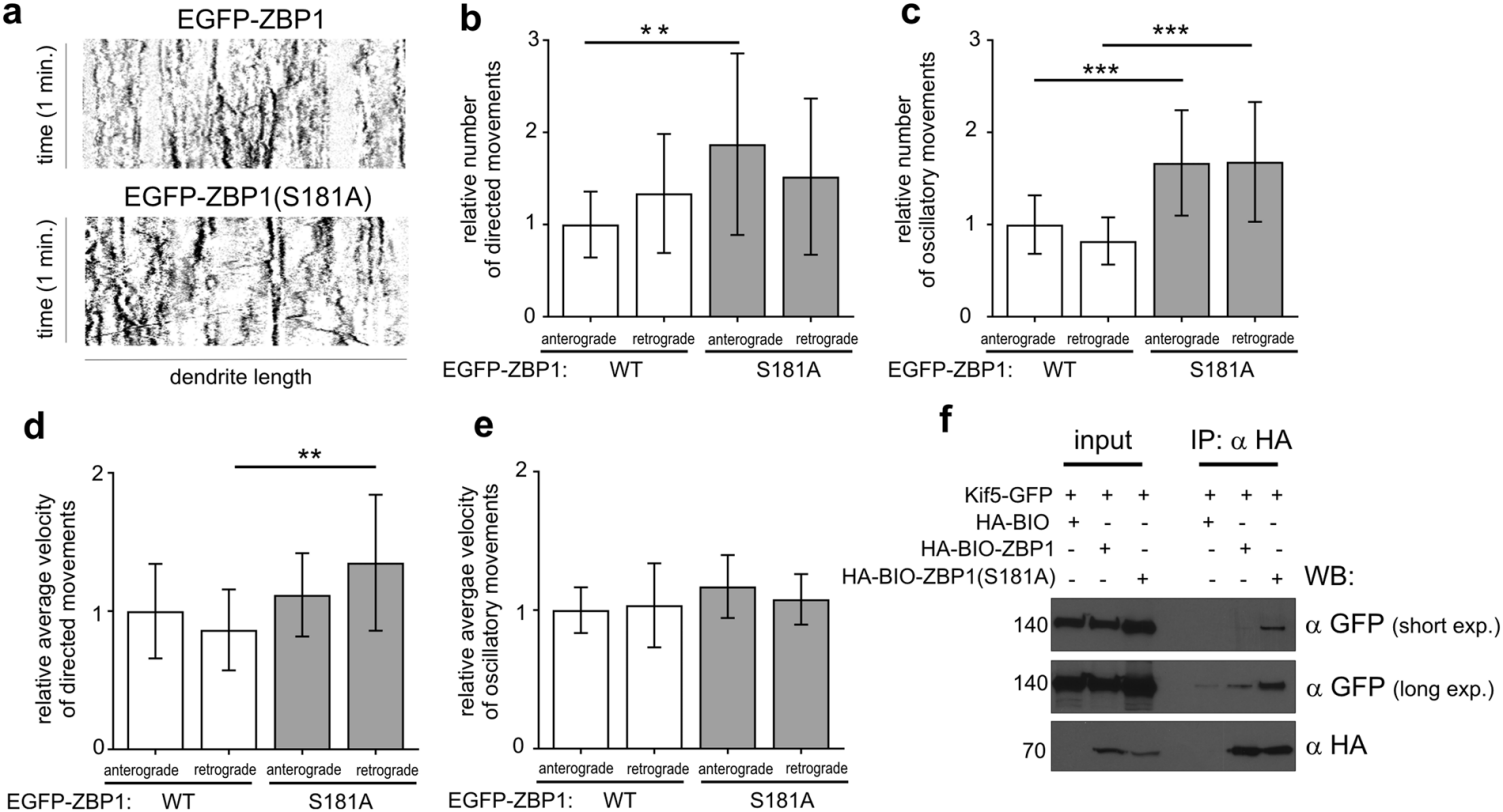
Non-phosphorylated ZBP1 granules are more motile. (**a**) Representative kymographs showing motility of EGFP-ZBP1 and EGFP-ZBP1_S181A_ granules in dendrites. (**b**,**c**,**d)** Relative numbers of ZBP1 and ZBP1_S181A_ granule movements. Granule movements were quantified, normalized per 10 μm of the dendrite length, and then normalized to the mean number of anterograde movements in the wildtype ZBP1 variant for each experiment, respectively. The data in the columns are expressed as a ratio to the mean number of movements. (**e)** Granule velocity was normalized to the mean anterograde velocity in the wildtype ZBP1 variant for each experiment, respectively. ***p < 0.001, *p < 0.1 (Student’s *t*-test). The data were analyzed from five neurons per group per experiment, with three independent experiments. Total raw number of directed anterograde or retrograde movements per group per experiment ranged from 15 to 57, and the number of oscillatory anterograde or retrograde movements per group per experiment ranged from 99 to 245 (for complete data see Table S1). The data are expressed as mean ± SD. (**f**) Western blot analysis of KIF5A-GFP bound to HA-BIO-ZBP1 or HA-BIO-ZBP1_S181A_ immunoprecipitated from Neuro2a cells transfected as indicated. Cropped blots are presented (for full length blots see Supplementary Information).

To examine whether the aberrant distribution of ZBP1_S181A_ results from a change in the motility of ZBP1-containing granules, we live-imaged neurons that overexpressed EGFP-ZBP1 or EGFP-ZBP1_S181A_. To visualize granule movement, we transfected DIV8-9 neurons with wildtype EGFP-ZBP1 or its S181A mutant and recorded time-lapse images 10–14 h after transfection. The movies were converted into kymographs, and all single movements were counted. All of the movements were divided into anterograde and retrograde, then classified as either directed (>2.5 µm) or oscillatory (<2.5 µm). The total number of movements per neuron was normalized per 10 µm of the dendrite and then to the mean number of anterograde movements in the wildtype variant in each experiment, respectively, to account for the variability of transfection. As shown in Fig. 5, ZBP1_S181A_ granules exhibited significantly more directed movements in the anterograde direction compared with the wildtype variant. At the same time, the velocity of retrograde directed movements was significantly elevated. In the case of the oscillatory movements, ZBP1_S181A_ granules were more motile in both directions, anterograde and retrograde. Consistent with this observation, we found more molecular motor KIF5A-GFP precipitated with ZBP1_S181A_ than with wildtype ZBP1 when both proteins were overexpressed in Neuro2a cells (Fig. 5f).

## Discussion

In the present study, we found that ZBP1 is stably phosphorylated in neurons at Ser181 in an mTORC2-dependent manner. We also provided evidence that S181 phosphorylation of ZBP1 is decreased in cells with increased Src activity, likely upon ZBP1 phosphorylation by Src. Further investigation of the functional significance of ZBP1 Ser181 phosphorylation in neurons revealed that this posttranslational modification contributed to proper dendritic arborization, dendritic ZBP1 distribution and mobility through the regulation of ZBP1 association with motor protein KIF5. We have also demonstrated that ZBP1 S181 phosphorylation is required, to some extent, for β-actin mRNA binding.

The first important finding of the present study was that in neurons, similar to non-neuronal cells, ZBP1 is phosphorylated at Ser181 in an mTORC2-dependent manner. Under basal conditions, this phosphorylation was stable. In fact, in neurons, the stability of Ser181 phosphorylation appears to be greater than in RD rhabdomyosarcoma cells^14^ because its decrease requires substantially longer mTORC2 inhibition. In RD cells and mouse embryonic fibroblasts (MEFs), Ser181 phosphorylation is protected by mRNA binding. Although not directly, our data support the existence of such a mechanism also in neurons. For example, we showed that ZBP1_GXXG_, which cannot bind mRNA, is poorly phosphorylated at S181. We also found that the overexpression of active Src decreased Ser181 ZBP1 phosphorylation. Tyr396 ZBP1 phosphorylation by Src results in the release of ZBP1-bound mRNA, and our data are consistent with the model that was proposed by Dai *et al*.^14^. Src-driven ZBP1 phosphorylation is locally and dynamically regulated in neurons by extracellular stimuli that control neuronal development (e.g., brain-derived neurotrophic factor)^6^. Thus, the present findings suggest that phosphorylation at Ser181 may undergo local dynamic regulation during different steps of neuronal development (e.g., axonal growth and dendritic arborization). However, as discussed below, our study does not address directly physiological consequences of such local and dynamic dephosphorylation of ZBP1.

Another important finding in the present study was that Ser181 ZBP1 phosphorylation is required for proper dendritic arborization. In contrast, Ser181 dephosphorylation does not appear to be as critical for this process, since ZBP1WT and ZBP1_S181E_ did not present different abilities to rescue the effects of endogenous ZBP1 knockdown. This observation is consistent with the hypothesis that the majority of cellular ZBP1 is phosphorylated at Ser181^14^. However, it raises a legitimate question, namely the way in which insufficient Ser181 ZBP1 phosphorylation disturbs dendritogenesis. Our previous study suggested that ZBP1 contributes to dendritic growth to ensure the accuracy of local β-actin translation^9^. While our results from Neuro2a cells confirm that the S181A mutant binds less β-actin mRNA, this difference did not show complete loss of function. Additionally, our preliminary results did not reveal differences in β-actin mRNA colocalization with wildtype ZBP1 and its Ser181 mutant that were overexpressed in hippocampal neurons (Supplementary Fig S7 and data not shown). Finally, both wildtype ZBP1 and its Ser181 mutants were comparably phosphorylated by Src. Thus, while we cannot exclude that both β-actin mRNA binding and release are influenced by a lack of Ser181 phosphorylation, the effect is not as striking and the altered phenotype of the dendritic tree is not likely to be a result of it. Dai *et al*.^14^ suggested that Ser181 ZBP1 phosphorylation is essential for the initiation of IGF2 mRNA translation using an internal ribosome entry site (IRES). Thus, one possibility is that in neurons that lack Ser181-phosphorylated ZBP1, only the IRES-driven translation of a subset of proteins that are needed for dendritogenesis is decreased. The regulation of IGF2 translation in neurons and the impact of IGF2 on dendritic arborization remain unknown. Several mRNAs that are transported and locally translated in dendrites can utilize IRES-dependent translation^18–20^. Among these are tropomyosin receptor kinase B (TrkB), fragile X mental retardation protein (FMRP1), microtubule associate protein 2 (MAP2), and Ca^2+^/calmodulin-dependent protein kinase II (CamKIIα), which are known regulators of dendritic arborization^21^. However, whether their IRES-dependent translation contributes to this process and can be controlled by ZBP1 remain unknown. Finally, we previously found that during intensive dendritogenesis, unlike in more mature neurons, ZBP1 is not equally distributed and is concentrated at dendritic branching points^9^, suggesting a functional link between this specific distribution pattern and dendritic growth. In the present study, ZBP1_S181A_ failed to concentrate at dendritic branching points and was more evenly distributed along dendrites, thus resembling the distribution of ZBP1 in neurons that passed the dendritogenesis stage. We hypothesize that Ser181 phosphorylation ensures the accurate spatial distribution of ZBP1 that is needed for efficient dendritic growth and branching. Ser181 phosphorylation may promote enrichment of ZBP1 at dendritic branch points and augment the locally translated pool of β-actin mRNA. Previous work has shown that axonal synthesis of β-actin supports axon branching of sensory neurons *in vitro* and *in vivo* ^22^. It has also been demonstrated that axonal branch points are hotspots of local protein synthesis, and they are enriched in ZBP1 and stalled mitochondria, the latter one being critical for local protein synthesis^23^. Curiously, in addition to ZBP1, dendritic branch points are also enriched in mitochondria^24^ as well as translational machinery^25^, suggesting some parallels between dendrite and axon branch points. Therefore, while the role of potential protein synthesis in dendritic branch points remains unclear and ZBP1 S181 phosphorylation in axonal branching has not been investigated, the results of our study suggest a potential mechanisms and provide motivation for future work on shared mechanisms between the dendrite and axon.

How does Ser181 phosphorylation regulate proper dendritic ZBP1 distribution? In this work, we found that ZBP1_S181A_ moved more than its wildtype counterpart. The greatest difference was observed in the number of movements, whereas the average velocity of the movements of the non-phosphorylated mutant was significantly greater but not as striking. This essentially means that ZBP1_S181A_-containing granules do not stop as often, suggesting that the increase in motility in the S181A variant was attributable to a disturbance in the mechanism that is responsible for granule docking (Supplementary Fig. S7). Consistent with this, a greater proportion of ZBP1_S181A_-containing granules remained mobile and bound to KIF5A. MyoVa is one example of a protein that can effectively prevent ZBP1 motility in axons^26^. Inhibition of MyoVa resulted in release of ZBP1 from actin and increases motility on microtubules^26^. Thus, lack of Ser181 phosphorylation may decrease its anchoring to actin at branch points. Alternatively, lack of Ser181 phosphorylation could directly increase its interaction with microtubule motors for transport in the dendrite (e.g., KIF5A, dynein, and KIF11)^27, 28^ and/or interactions with other scaffolds (e.g., huntingtin and HAP1)^28^, leading to lower capability of ZBP1 to enter stationary state at the correct destination. Regardless of the exact molecular mechanism enforcing more uniform ZBP1_S181A_ distribution in dendrites, loss of proper ZBP1 localization likely impairs the discrete pattern of local protein synthesis, required for proper dendritogenesis.

The present work focused on the role of ZBP1 S181 phosphorylation by examining the consequences of its absence. Our results indicate that phosphorylation of ZBP1 at S181 is needed for its full capacity to bind β-actin mRNA and accurate mobility of ZBP1-containing granules (or their ability to enter a stationary state needed for proper local mRNA storage; Supplementary Fig. S7). In fact, it is possible that improper ZBP1 mobility results from improper granule formation and/or mRNA loading. However, we can not exclude that these two phenomena are independent. Although S181 phosphorylation is quite stable, our data imply that under some circumstances (e.g. Y396 phosphorylation by Src, RNA release) this serine may undergo physiological dephosphorylation. However, due to the nature of our approach we can only speculate what could be the role of it. Since this dephosphorylation likely occurs as a result of RNA release, which ends the life cycle of a granule, it could simply be the outcome of the disintegration of the granule. Nevertheless, it is also possible that the changed mobility and RNA binding capability, is a result of the dephosphorylation, and it prevents ZBP1, from immediate resilencing of newly released mRNA (Supplementary Fig. S7). However, further study is needed to address the physiological role of dephosphorylation of ZBP1 S181.

## Methods

### DNA constructs

The following plasmids were described previously: pGW1-HA^29^, pSuper^30^, pSuper-shRaptor#1^16^, pSuper-shRictor#2^16^, pEGFP-ZBP1^8^, pSuper-ZBP1sh#1^9^, pEGFP-ZBP1_GXXG_^9^, pEGFP-ZBP1_Y396F_^9^, pEGFP-ZBP1*1^9^, β-actin-mRFP^31^, pSGT-Src^5^, pSGT-Src_CA_(K239Q, P250G)^5^, pcDNA-Src_KD_(Y416F)^5^, pEGFPC2-BIO^32^, HA-BirA^32^, and tdTomato^33^. pEGFP-ZBP1-P2A-tdTomato was constructed based on pEGFP-ZBP1, in which the GFP stop codon was exchanged with sequences that encoded the P2A signal followed by tdTomato. The change of ZBP1 (or ZBP1*1) Ser181 to alanine (A) or glutamic acid (E) was performed using the Quick Change Site Mutagenesis System (Stratagene, La Jolla, CA, USA). To change Ser181 to A, the following primers were used: 5′-CCCAGGCAGGGGGCACCGGTAGCAG-3′ (forward) and 5′-CTGCTACCGGTGCCCCCTGCCTGGG-3′ (reverse). The following primers were used for S181E substitution: 5′-CCCAGGCAGGGGGAACCGGTAGCAG-3′ (forward) and 5′-CTGCTACCGGTTCCCCCTGCCTGGG-3′ (reverse). Substitution of Y396 to F was performed as described previously^9^. Bio-Thrombin-HA plasmid was generated by changing the GFP coding sequence of pEGFPC2-BIO to a sequence that encoded the thrombin recognition site followed by an HA tag and was a kind gift from Dr. Swiech. Next, Bio-Thrombin-HA-ZBP1 and its mutated derivatives were constructed by the polymerase chain reaction (PCR) subcloning of the ZBP1 (or its mutants) coding sequence to EcoRI/SalI sites of Bio-Thrombin-HA. pGW1-Src^WT^-HA, pGW1-Src^CA^-HA, and pGW1-Src^KD^-HA were obtained by subcloning cDNA-encoding Src and its mutants that were obtained by PCR (5′-GCAGATCTATGGGGAGCAGCAAGAGCAAGC-3′ and 5′-GGGAATTCCTATAGGTTCTCTCCAGGCTGG-3′) in BglII/EcoRI sites of the GW1-HA plasmid. β-actin-KIF5A-GFP plasmid was a kind gift from Drs. Lipka and Hoogenraad.

### Antibodies and drugs

The commercially available primary antibodies that were used in the present study are listed in Table 1. Rabbit anty-P-ZBP1(Tyr396) antibody was described previously^6^. Cross-affinity purified rabbit polyclonal anti-ZBP1(S181) was produced by Eurogentec (Liege, Belgium) against PRQGS*PVAAGA peptide. Both were used at a 1:100 dilution for Western blot. Horseradish peroxidase-conjugated secondary antibodies and IRDye^®^ 680RD anti-mouse and IRDye^®^ 800CW were obtained from Jackson ImmunoResearch (West Grove, PA) and LI-COR Biosciences (Lincoln, NE), respectively. INK128 was purchased from Selleckchem (Munich, Germany). Cycloheximide and alkaline phosphatase (FastAP), were obtained from Sigma-Aldrich (St. Louis, MO) and Thermo Scientific (Waltham, MA), respectively.

**Table 1:** Primary antibodies used for the study.

Antigen	Manufacturer	Cat. number	Host	Application
Akt	Cell Signaling Technology Danvers, MA	4298	mouse	WB 1:2000
phospho-Akt (Ser473)	Cell Signaling Technology,	4060	rabbit	WB 1:1000
β-actin	Sigma-Aldrich, Saint Louis, MO	A2228	mouse	IF 1:1000
GAPDH	Synaptic Systems, Goettingen, Germany	247002	rabbit	WB 1:1000
GAPDH	EMD Millipore, Billerica, MA	MAB374	mouse	WB 1:1000
GFP	MBL, Woburn, MA	598	rabbit	IF 1:1000 WB 1:3000
GFP	Sigma Aldrich	11 814 460 001	mouse	RNA-IP: 15 µg/sample
HA	Sigma-Aldrich	11 867 423 001	rat	WB 1:1000
IGF2BP1 (ZBP1)	MBL	RN007P	rabbit	WB 1:1000
IGF2BP1 (ZBP1)	MBL	RN001M	mouse	WB 1:1000
IMP1 (ZBP1)	SantaCruz Biotechnology, Dallas, TX	21026	goat	WB 1:1000
IMP1 (ZBP1)	Cell Signaling Technology	8482	rabbit	WB 1:1000
Raptor	Cell Signaling Technology	2280	rabbit	WB 1:1000
Rictor	Cell Signaling Technology	2114S	rabbit	WB 1:1000
S6	Cell Signaling Technology	2217	rabbit	WB 1:1000
phospho-S6 (Ser235/236)	Cell Signaling Technology	4858L	rabbit	WB 1:1000
v-Src	EMD Millipore, Billerica, MA	OP07	mouse	WB 1:1000
mRFP (tdTomato)	Evrogen, Moscow, Russia	AB233	rabbit	WB 1:1000
α-tubulin	Sigma-Aldrich	T5168	mouse	WB 1:5000

WB - Western blot, IF – immunofluorescence.

### Primary neuron cultures and transfection

Primary cultures of rat hippocampal and cortical neurons were prepared as described previously^9^ in accordance with the institutional guidelines of the First Local Ethics Committee in Warsaw (decision for experimental protocol no. 205/2011), which are in compliance with the European Community Council Directive (86/609/EEC). Cells were transfected on the indicated DIV using Lipofectamine2000 or nucleofected on DIV0 as described previously^9^. Primary cultures of hippocampal neurons of *IMP1* ^−/−^ mice were prepared from E17 embryos obtained by crossing *IMP1* ^*+*/*−*^ mice as described^34^ and in accordance with the institutional guidelines of Emory University (protocol approved by Emory University IACUC committee [GJB]). DIV7 cells were transfected using Lipofectamine2000.

### Neuro2A culture, transfection and drug treatment

Neuro2a cells (ATCC, Manassas, VA, USA) were grown in Dulbecco’s Modified Eagle Medium that contained 10% fetal bovine serum and 1% penicillin-streptomycin (all from Sigma-Aldrich) at 37 °C in a 5% CO_2_ atmosphere. The cells were transfected using JetPEI (PolyPlus, Illkirch, France) at a 3:1 PEI:DNA ratio according to the manufacturer’s instructions. Eighteen hours after transfection, the cells were treated with cycloheximide (10 μg/ml) for 0.5, 1, 3, 6 or 24 hours. The control cells were treated with vehicle (98% ethanol).

### *In vitro* phosphatase assay

*In vitro* phosphatase assay is described in Supplementary Materials and Methods.

### Avi-tag pulldown and immunoprecipitation

Biotinylated forms of proteins were produced in the Neuro2a cell line. These cells were transfected with a plasmid that encoded ZBP1 or its mutants with an avi-tag and BirA plasmid that encoded the biotin ligase^32^. Eighteen hours after transfection, the cells were placed in ice-cold PBS, centrifuged at 8,000 × *g* for 3 min, and lysed for 10 min in lysis buffer (20 mM Tris [pH 7.5], 100 mM NaCl, and 0.5% NP-40) supplemented with protease and phosphatase inhibitors. The cell lysate was clarified by centrifugation at 18,000 × *g* at 4 °C for 20 min and incubated for 1.5 h at 4 °C with streptavidin-coated magnetic beads (Dynabeads M-280, Invitrogen). After incubation, the beads were washed five times with lysis buffer, and bound proteins were recovered by boiling the samples with 1× Laemmli buffer.

For immunprecipitation Neuro2a cells were transfected with plasmids encoding Kif5a-GFP and Bio-HA (negative control), ZBP1-Bio-HA, or ZBP1_S181_-Bio-HA. Forty-eight hours after transfection, the cells were collected in ice-cold TBS-K (20 mM Tris [pH 7.5], 100 mM NaCl), centrifuged at 4,000 × *g* for 10 min, and lysed for 20 min in lysis buffer (50 mM Tris [pH 7.5], 150 mM NaCl, 0,1% Triton X-100, 2 mM EDTA, 2 mM MgCl_2_) supplemented with 2 mM ATP. The cell lysate was cleared by centrifugation at 13,000 × g at 4 °C for 20 min and incubated overnight with magnetic beads covered with anti-HA antibody (Pierce™ Anti-HA Magnetic Beads, ThermoFisher). After incubation, the beads were washed four times with lysis buffer without ATP, and boiled in 1 × Laemmli buffer.

### RNA co-immunoprecipitation

Immunoprecipitation was performed as before, with modifications (Janusz *et al*., 2013)^35^. Briefly, Neuro2A cells were transfected with EGFP-ZBP1 or EGFP-ZBP1_S181A_ plasmid. Next day, cells were collected, washed with PBS and suspended in 1 ml of a buffer containing 140 mM KCl, 1.5 mM MgCl_2_, 20 mM Tris-HCl (pH 7.4), 0.5% Nonidet P-40, 0.5 mM dithiothreitol, 100 U/ml RiboLock (Thermo Scientific) and protease inhibitors. Extracts (800 µg of total protein) were precleared with 60 µl of Dynabeads Protein G (Invitrogen) for 2.5 h. Afterward, 1/10 of each supernatant was saved as input fraction for WB and RNA isolation. Protein-RNA complexes were precipitated overnight in 4 °C with 60 µl of Dynabeads Protein G, conjugated with either mouse anti-GFP antibody (Sigma-Aldrich) or normal mouse IgG (Sigma-Aldrich). Next, 1/6 of the beads was collected and boiled with 1× Laemmli buffer for WB. The remaining beads were suspended in 500 µl TRIzol (Thermo Scientific) for 5 min. Then, TRIzol was collected and RNA was isolated. For the quantitative real-time (qRT)-PCR, RNA was suspended in 11 µl of H_2_O, then its concentration was determined with NanoDrop (Thermo Scientific). Reverse-transcription was performed with High Capacity cDNA Reverse Transcription Kit (Applied Biosystems, Foster City, CA), according to manufacturer’s instruction.

### Quantitative real time PCR

For the RT-qPCR, Taqman probes for β-actin and the control gene USP16 were used (Mm01344526_m1, Mm01205647_g1; Applied Biosystems). Fold enrichment in the IP fraction was calculated for both mRNAs according to the standard curve method, using Input ZBP1 WT cDNA as a standard. Results were corrected for the non-specific background in the IgG fraction and ratios of β-actin and USP16 mRNA were calculated. To account for the difference in the transfection levels ratios were normalized to the ZBP1 WT IP fraction.

### Western blot

Qualitative and quantitative Western blot analyses were performed as described previously^36^.

### Imaging of fixed cells and image analysis

The acquisition of confocal images of fixed neurons and morphometric analysis were performed as described previously^9^. Dendritic distribution of overexpressed EGFP-ZBP1 and its mutants was analyzed as previously^9^ with additional normalization step. Briefly, images of cells were obtained using a Zeiss LSM710 NLO laser confocal microscope with a 100 × 1.3 objective. Images were acquired in z-series (0.5 µm steps), with identical acquisition settings for all neurons. For analysis, z-series were rendered using maximum intensity projections, exported into TIFF format, and analyzed using MetaMorph software. Profiles of EGFP-ZBP1 and TdTomato fluorescence along the dendrites were made using the Linescan function, with the scan width set to 6 pixels. For each pixel EGFP fluorescence was standardized to fluorescence of TdTomato. Next, branching sites were indicated manually, based on the TdTomato imaging of dendrites and the mean standardized intensities of EGFP fluorescence at (R segments) and between (D segments) the branching sites were calculated using OpenOffice Calc software. D and R segments were not of the same length or area.

### Live imaging of hippocampal neurons in culture

For live imaging, rat primary hippocampal neurons were cultured on glass-bottom multi-well plate dishes (12-well, no. 1.0 coverslip, 14 mm glass diameter; MatTek, Ashland, MA, USA), pre-cleaned with 1 M HCl according to the manufacturer’s specifications, and then coated with laminin and poly-L-lysine^9^. Neurons were transfected on DIV7-8 with pEGFP-ZBP1 or pEGFP-ZBP1_S181A_. Time-lapse images (135 frames in 0.444 s intervals) of neurons that expressed EGFP-ZBP1 or EGFP-ZBP1_S181A_ were collected 10-14 h after transfection. Image analysis was performed using ImageJ software. All stack images were first corrected for X-Y drift. Afterward, to remove immobile background foci, the averaged image was subtracted from the image stack. The region of interest (ROI) for all dendrites in focus that were longer than 15 µm were converted to kymographs (x = dendrite length, y = time). All single movements were counted and divided into anterograde or retrograde based on their x-y slope, then classified as either directed (>2.5 µm) or oscillatory (<2.5 µm).

### Statistical analysis

The exact numbers of cells (*n*) and culture batches (*N*) that were examined and the statistical tests that were used for the respective experiments are indicated in the figure legends. The statistical analyses were performed using Prism software (GraphPad, San Diego, CA).

## Electronic supplementary material

Supplementary Information

Supplementary Table S1
