# Supplementary material for: ZBP1 phosphorylation at serine 181 regulates its dendritic transport and the development of dendritic trees of hippocampal neurons
Source: Sci Rep. 2017 May 12;7:1876. doi: 10.1038/s41598-017-01963-2 (PMC5431813; doi:10.1038/s41598-017-01963-2)
Supplement: Supplementary file 1 — Supplementary Information [file 41598_2017_1963_MOESM1_ESM.pdf]

Supplementary Information

ZBP1 phosphorylation at serine 181 regulates its dendritic transport and the development of dendritic trees of hippocampal neurons

Anna S. Urbanska, Aleksandra Janusz-Kaminska, Katarzyna Switon, Alicia L. Hawthorne, Malgorzata Perycz, Malgorzata Urbanska, Gary J. Bassell & Jacek Jaworski

1. Supplementary Figures

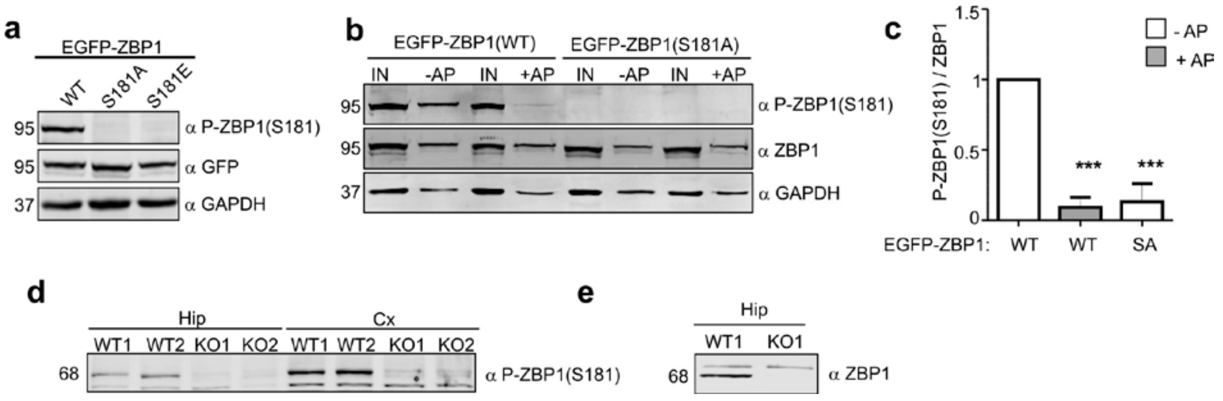

Supplementary Fig. S1. Anti-P-ZBP1(S181) antibody is specific. a, Western blot analysis of total levels of EGFP-ZBP1 phosphorylated at Ser181 (P-ZBP1[S181]) and EGFP-ZBP1 (with GFP antibody) in protein lysates obtained from Neuro2a cells transfected with pEGFP-ZBP1, pEGFP-ZBP1$_{S181A}$, and pEGFP-ZBP1$_{S181E}$. GAPDH is shown as an additional loading control. Cropped blots are presented (for full length blots see below). **b,** Western blot analysis of levels of ZBP1 phosphorylated at Ser181 (P-ZBP1[S181]) and total ZBP1 in protein lysates obtained from Neuro2a cells transfected with pEGFP-ZBP1, pEGFP-ZBP1S181A and treated with alkaline phosphatase (AP). GAPDH is shown as an additional loading control. Cropped blots are presented (for full length blots see below). **c,** Results of quantitative Western blot analysis. The data are presented as a ratio of the signal intensity of P-ZBP1(S181) to the signal intensity of ZBP1 ± SEM. ***p < 0.001 (One sample t-test). Number of independent experiments (N = 3). **d**, Western blot analysis of levels of ZBP1 phosphorylated at Ser181 (P-ZBP1[S181]) in protein lysates obtained from hippocampi and cortices of wildtype and E16 *IMP1*$^{-/-}$ mice. Cropped blots are presented (for full length blots see below). **e**, Western blot analysis of total levels of ZBP1 in protein lysates obtained from hippocampi and cortices of wildtype and E16 *IMP1*$^{-/-}$ mice. Cropped blots are presented (for full length blots see below).

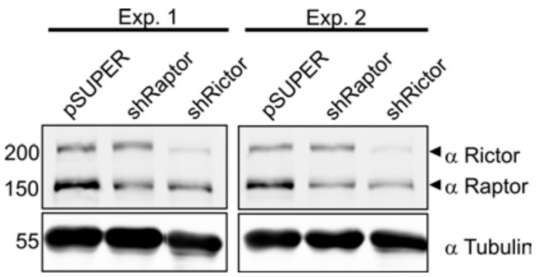

Supplementary Fig. S2. shRNAs against Rictor and Raptor work effectively in neurons. Western blot analysis of Raptor and Rictor levels in protein lysates obtained from DIV8 neurons nucleofected on DIV0 with empty pSuper (control vector) or pSuper that encoded shRNA against Raptor or Rictor. α-Tubulin is shown as an additional loading control (same experiments as in Fig. 1a). Cropped blots are presented (for full length blots see below).

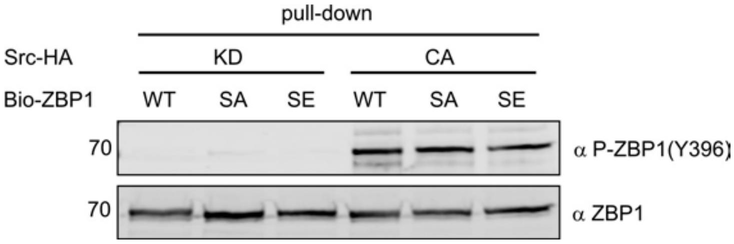

Supplementary Fig. S3. ZBP1 phosphorylation at Ser181 is not needed for its Tyr396 phosphorylation by Src. Western blot analysis of Tyr396 phosphorylation of Bio-HA-ZBP1 that was pulled down from Neuro2a cells transfected with pBio-ZBP1, pBio-ZBP1$_{S181A}$, or pBio-ZBP1$_{S181E}$ and plasmids that encoded kinase-dead (KD) and constitutively active (CA) mutants of Src. Cropped blots are presented (for full length blots see below).

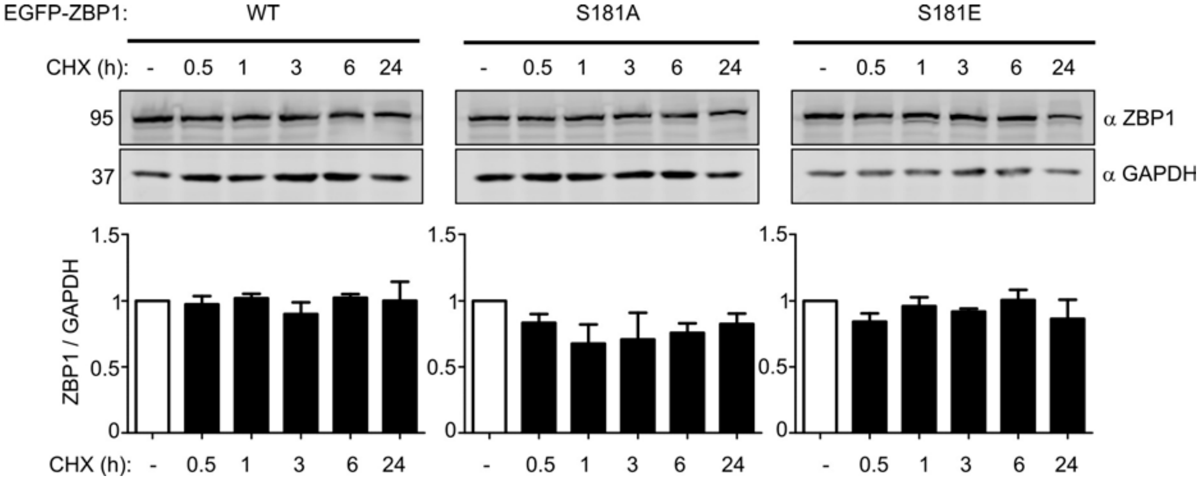

Supplementary Fig. S4. Mutations of ZBP1 Ser 181 do not affect ZBP1 stability in Neuro2a cells. *Upper panels*: Western blot analysis of EGFP-ZBP1 or its S181A and S181E mutant stability in transfected Neuro2a cells treated with cycloheximide (10 μg/ml) as indicated. GAPDH is shown as an additional loading control. Lower panels: Results of quantitative Western blot analysis. The data are presented as a ratio of the signal intensity of

ZBP1 to the signal intensity of GAPDH ± SEM. Cropped blots are presented (for full length blots see below).

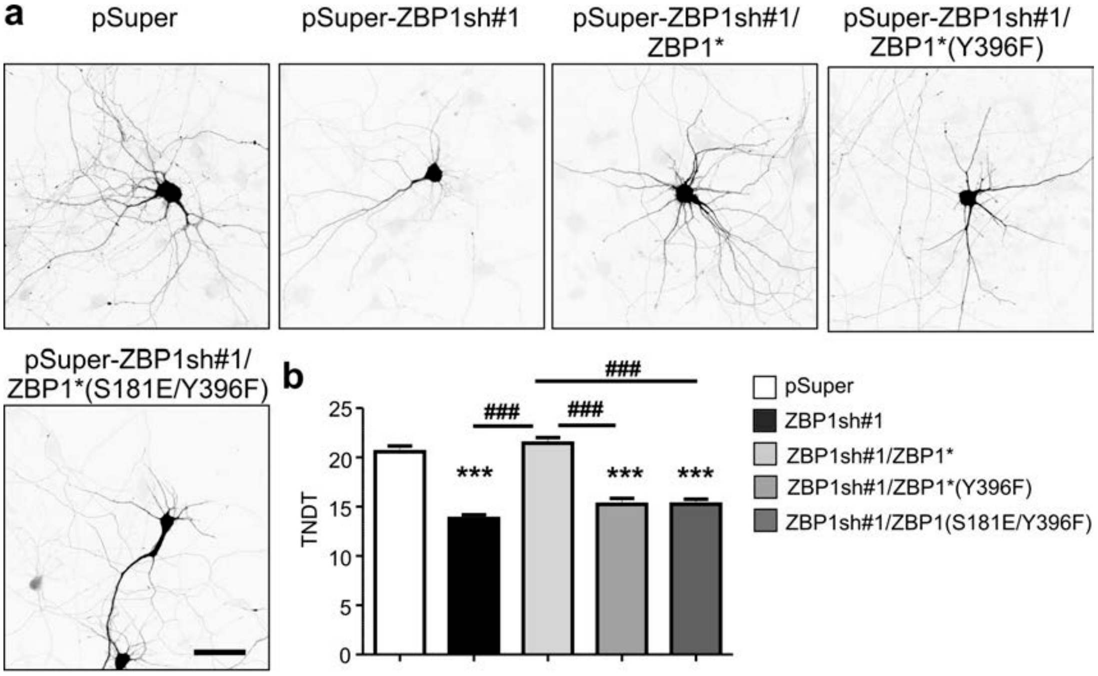

Supplementary Fig. S5. Lack of ZBP1 phosphorylation at Tyr396 cancels ZBP1$_{S181E}$ mutant ability to rescue ZBP1 shRNA-induced dendritic arborization phenotype of hippocampal neurons. a, Representative confocal images of cultured hippocampal neurons transfected on DIV8 for 2 days as indicated. Neuron morphology was visualized by co-transfection with mRFP. Scale bar = 50 µm. **b**, Total number of dendritic tips (TNDT) of neurons transfected as in **a**. The data are expressed as mean ± SEM. ***$p < 0.001$, compared with pSuper transfected cells; $^{###}p < 0.001$, compared as indicated (Kruskal-Wallis test followed by Dunn's *post hoc* test). Cell images were obtained from three independent culture batches. Number of cells per variant: pSuper (n = 63), ZBP1sh#1 (n = 65), ZBP1sh#1/pEGFP-ZBP1* (n = 60), ZBP1sh#1/pEGFP-ZBP1*$_{Y396F}$ (n = 61), and ZBP1sh#1/pEGFP-ZBP1*$_{S181E/Y396}$ (n = 59).

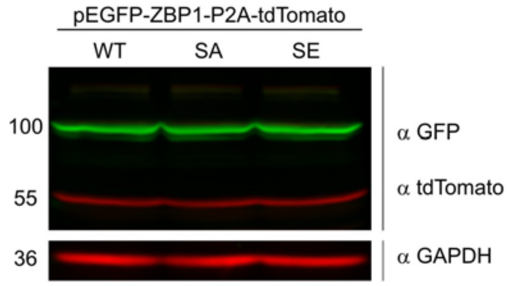

Supplementary Fig. S6. pEGFP-ZBP1-P2A-TdTomato allows simultaneous production of EGFP-ZBP1 variants and tdTomato. Western blot analysis of EGFP-ZBP1 (green) and tdTomato (red) levels in protein lysates obtained from Neuro2a cells transfected with pEGFP-

ZBP1-P2A-tdTomato, pEGFP-ZBP1$_{S181A}$-P2A-tdTomato, and pEGFP-ZBP1$_{S181E}$-P2A-tdTomato. GAPDH is shown as an additional loading control. Cropped blots are presented (for full length blots see below).

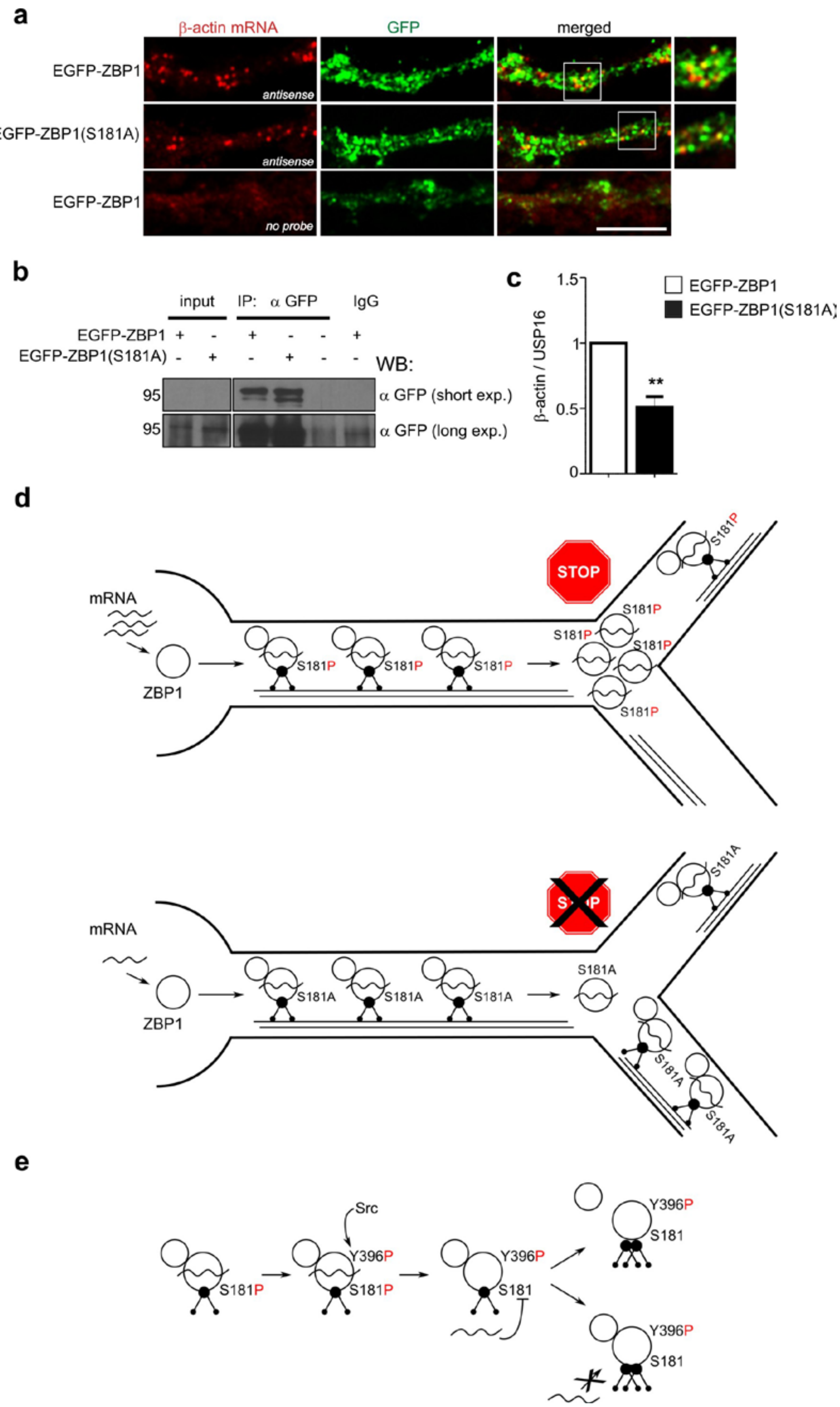

Supplementary Fig. S7. $ZBP1_{S181A}$ colocalizes with β-actin mRNA in dendrites and it is less enriched in β-actin mRNA in Neuro2A RNA-coIP. a, Microphotographs of dendritic segments of DIV7+3 hippocampal neurons derived from *IMP1*$^{-/-}$ mouse embryos transfected as indicated stained for β-actin mRNA (in situ hybridization, red) and GFP (IF, green). In situ hybridization was performed with Stellaris β-actin mRNA probe as described.[1] Images were obtained and processed the images with Zeiss LSM800 Airyscan. Scale bar = 5 µm. **b,** Western blot analysis of levels of EGFP-ZBP1 or EGFP-$ZBP1_{S181A}$ immunopreciptated with α-GFP antibody from Neuro2a cells transfected as indicated during RNA-IP procedure. Cropped blots are presented (for full length blots see below). **c,** Results of RT-qPCR analysis of enrichment in β-actin over USP16 (control) mRNA gene in the EGFP-ZBP1 and EGFP-$ZBP1_{S181A}$ IP. Data are presented as ratio of β-actin to USP16 mRNA in the EGFP-S181A variant normalized to the β-actin/USP16 EGFP-Zbp1 co-IP ± SEM. $^{**}p < 0{,}01$; One-sample *t*-test, (N=3). The results were corrected for unspecific binding in the IP IgG fraction. **d,** Proposed model of a role of ZBP1 S181 phosphorylation. When S181 is phosphorylated under physiological conditions, mRNA is properly loaded to the granules and transported to dendritic branching points where it is docked and stored until needed. This results in uneven ZBP1 granule distribution with enrichment in dendritic branch points. However, ZBP1S181A cannot recognize docking signals so it passes branch points, which eventually leads to a more even distribution of ZBP1 granules. **e,** Proposed model of a physiological role of ZBP1 S181 dephosphorylation. Although S181 phosphorylation is quite stable, upon Y396 phosphorylation by Src and RNA release this serine may undergo dephosphorylation. Such dephosphorylation could simply be the outcome of the disintegration of the granule or a mechanism preventing ZBP1from immediate resilencing of newly released mRNA (either due to lowered RNA binding capacity and/or increased motility).

1. Ifrim, M. F., Williams, K. R. & Bassell, G. J. Single-Molecule Imaging of PSD-95 mRNA Translation in Dendrites and Its Dysregulation in a Mouse Model of Fragile X Syndrome. *J. Neurosci.* **35,** 7116–7130 (2015).

2. Supplementary Materials and Methods

In vitro phosphatase assay

Neuro2a cells were transfected with plasmids encoding EGFP-ZBP1 or EGFP-ZBP1-S181A. Eighteen hours after transfection, the cells were collected in ice-cold TBS-K (20 mM Tris [pH 7.5], 100 mM NaCl), centrifuged at 4,000 x *g* for 10 min, and lysed for 20 min in lysis buffer (50 mM Tris [pH 7.5], 100 mM NaCl, 0.5% NP-40, 5 mM MgCl2), supplemented with either protease and phosphatase inhibitors, or protease inhibitors alone. The cell lysate was cleared by centrifugation at 13,000 x *g* at 4°C for 10 min. The input fraction (“IN”) was collected after centrifugation, and alkaline phosphatase (FastAP, 10 U/ 20 µg of protein lysates) was added to the lysates containing only protease inhibitors. The lysates were incubated in 37°C for 30 minutes, and the reaction was stopped by adding 4x Laemmli buffer.

3. Uncropped gel images

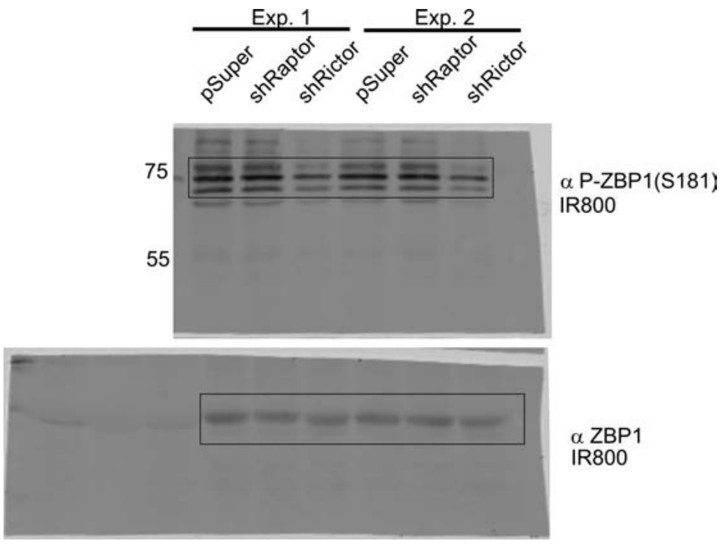

Fig. 1a.

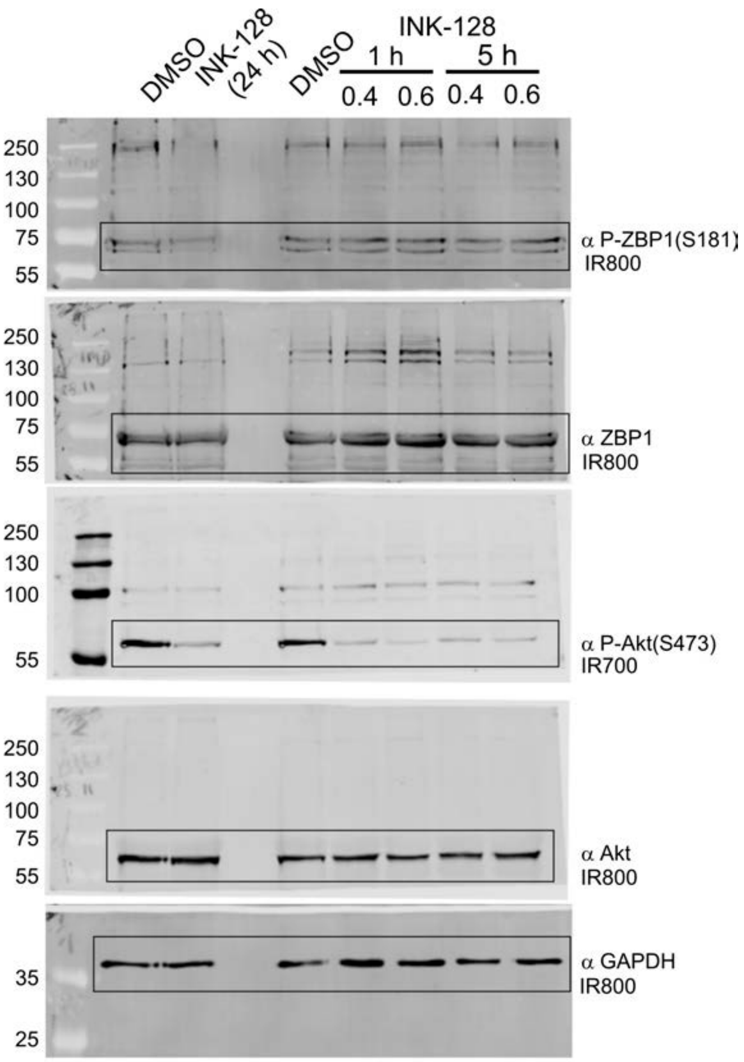

Fig. 1c

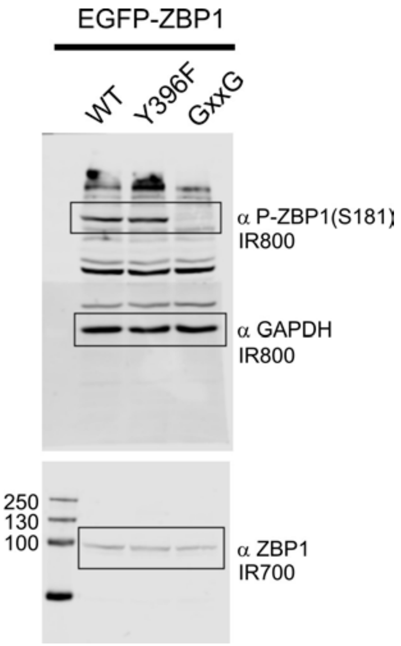
EGFP-ZBP1
WT
Y396F
GxxG
α P-ZBP1(S181)
IR800
α GAPDH
IR800
250
130
100
α ZBP1
IR700

Fig. 2a

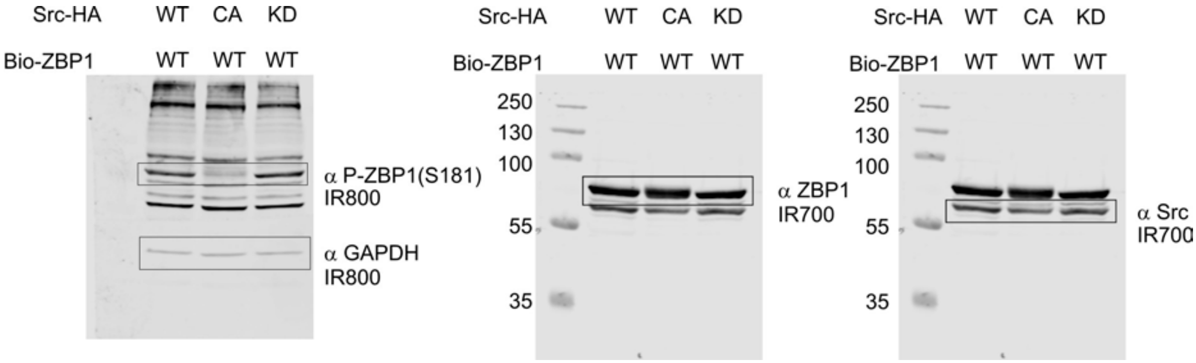
Src-HA WT CA KD
Bio-ZBP1 WT WT WT
α P-ZBP1(S181)
IR800
α GAPDH
IR800
Src-HA WT CA KD
Bio-ZBP1 WT WT WT
250
130
100
55
35
α ZBP1
IR700
Src-HA WT CA KD
Bio-ZBP1 WT WT WT
250
130
100
55
35
α Src
IR700

Fig. 2c

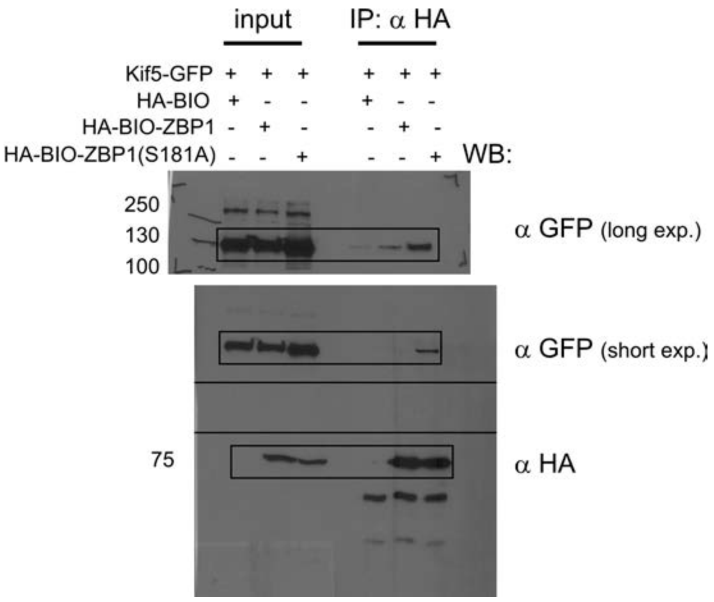

Fig.5f

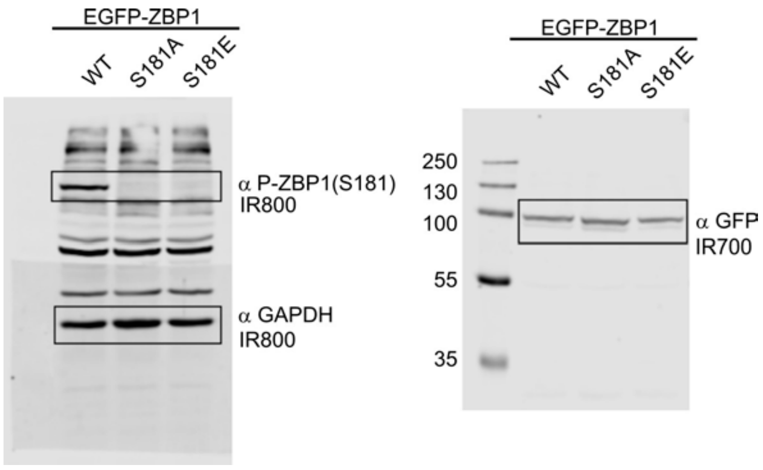

Supplementary Fig. S1a

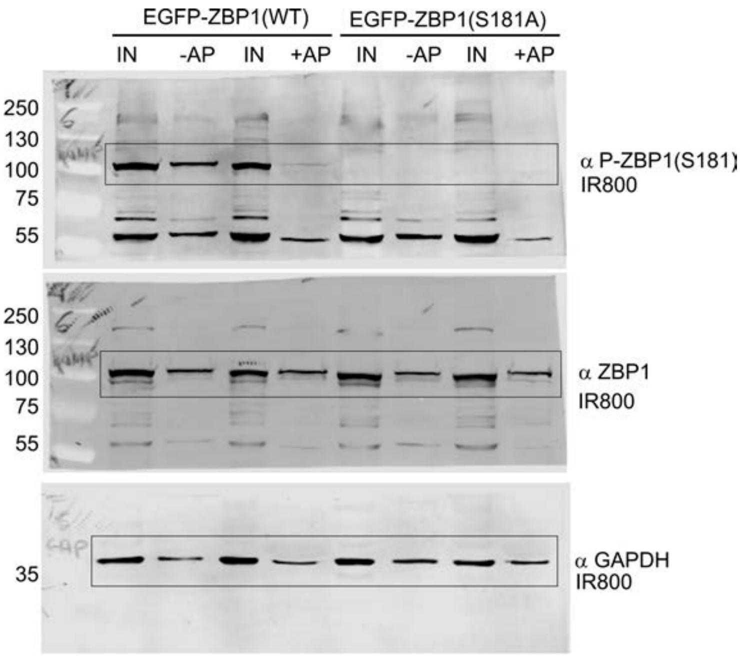

Supplementary Fig. S1b

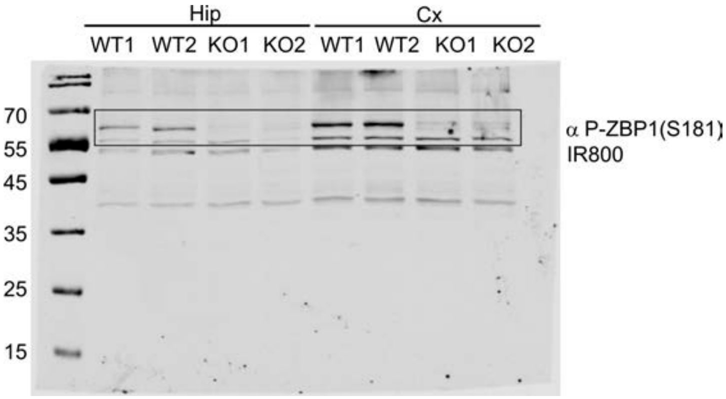

Supplementary Fig. S1d

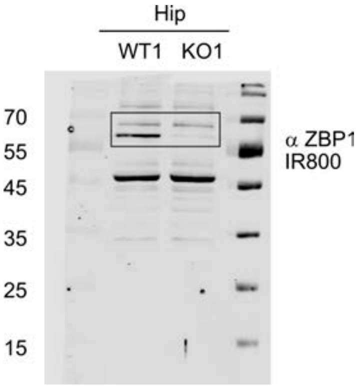

Supplementary Fig. S1e

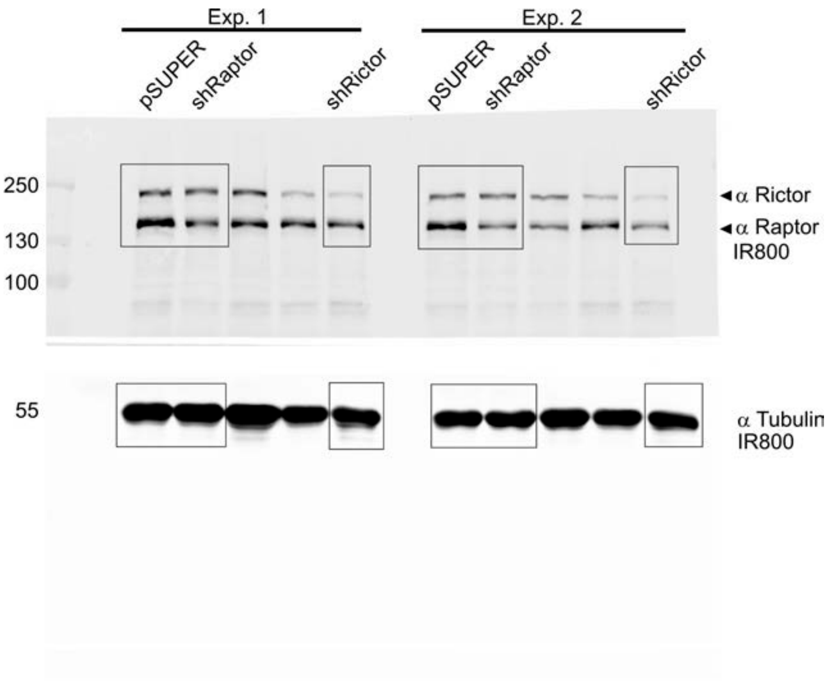

Supplementary Fig. S2

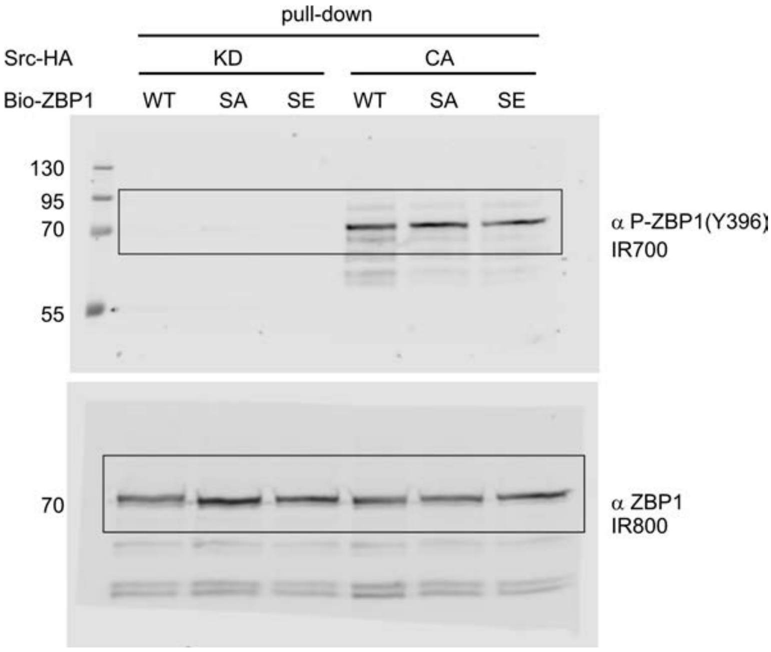

Supplementary Fig. S3

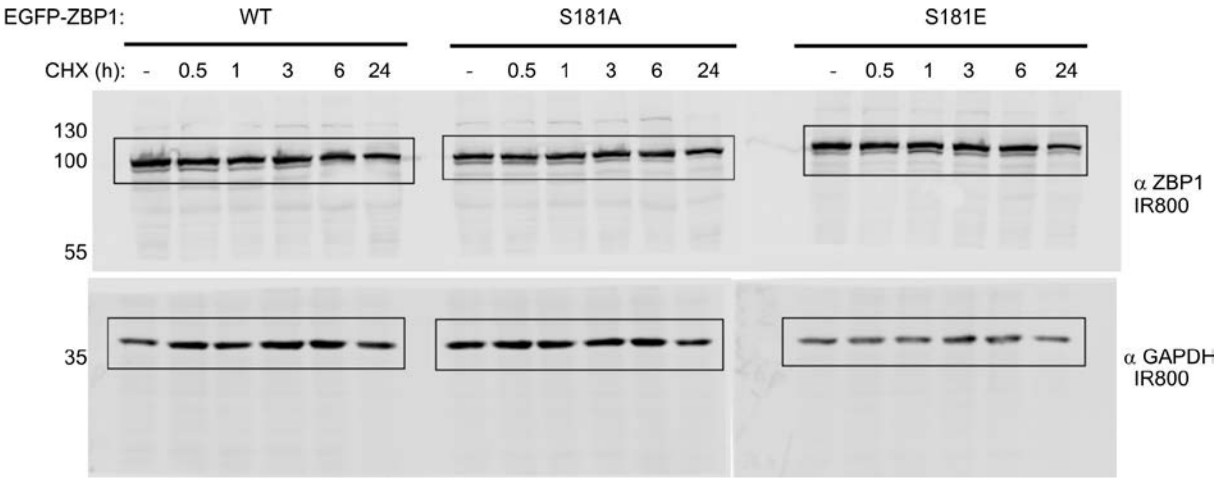

Supplementary Fig. S4

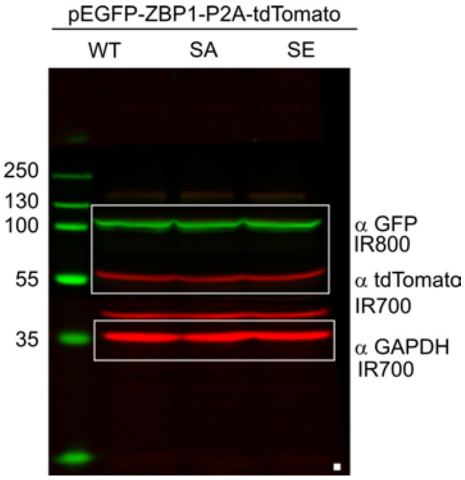

Supplementary Fig. S6

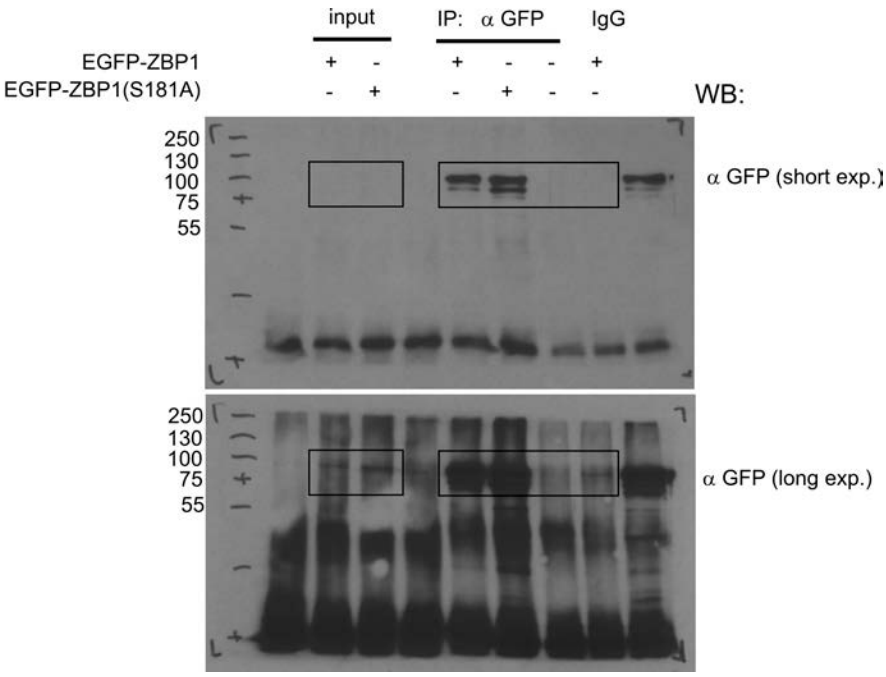

Supplementary Fig. S7
